# Phenethyl Isothiocyanate, a Dual Activator of Transcription Factors NRF2 and HSF1

**DOI:** 10.1002/mnfr.201700908

**Published:** 2018-06-19

**Authors:** Sharadha Dayalan Naidu, Takafumi Suzuki, Masayuki Yamamoto, Jed W. Fahey, Albena T. Dinkova‐Kostova

**Affiliations:** ^1^ Cullman Chemoprotection Center Johns Hopkins University Baltimore MD 21205 USA; ^2^ Department of Pharmacology and Molecular Sciences Johns Hopkins University School of Medicine Baltimore MD 21205 USA; ^3^ Department of Medical Biochemistry Tohoku University Graduate School of Medicine Sendai 980‐8575 Japan; ^4^ Department of Medicine Division of Clinical Pharmacology Johns Hopkins University School of Medicine Baltimore MD 21205 USA; ^5^ Department of International Health Center for Human Nutrition Johns Hopkins University Bloomberg School of Public Health Baltimore MD 21205 USA; ^6^ Jacqui Wood Cancer Centre Division of Cancer Research School of Medicine University of Dundee Dundee DD1 9SY Scotland UK

**Keywords:** HSF1, HSP90, KEAP1, NRF2, PEITC

## Abstract

Cruciferous vegetables are rich sources of glucosinolates which are the biogenic precursor molecules of isothiocyanates (ITCs). The relationship between the consumption of cruciferous vegetables and chemoprotection has been widely documented in epidemiological studies. Phenethyl isothiocyanate (PEITC) occurs as its glucosinolate precursor gluconasturtiin in the cruciferous vegetable watercress (*Nasturtium officinale*). PEITC has multiple biological effects, including activation of cytoprotective pathways, such as those mediated by the transcription factor nuclear factor erythroid 2 p45‐related factor 2 (NRF2) and the transcription factor heat shock factor 1 (HSF1), and can cause changes in the epigenome. However, at high concentrations, PEITC leads to accumulation of reactive oxygen species and cytoskeletal changes, resulting in cytotoxicity. Underlying these activities is the sulfhydryl reactivity of PEITC with cysteine residues in its protein targets. This chemical reactivity highlights the critical importance of the dose of PEITC for achieving on‐target selectivity, which should be carefully considered in the design of future clinical trials.

## 1 Introduction

Glucosinolates (β‐thioglucoside *N*‐hydroxysulfates; GS) are naturally occurring compounds that are precursors for isothiocyanates (ITCs). These phytochemicals are of widespread occurrence in the cruciferous vegetables, the Cruciferae or Brassicaceae family containing hundreds of genera and thousands of species, and about 15 other plant families.^1^ Over the past five decades it has been demonstrated in various preclinical, clinical, and epidemiological studies that dietary intake of cruciferous vegetables reduces the risk of cancer. In addition, many of the ITCs exert protective effects in neurodegeneration, cardiovascular diseases, bacterial infection, and inflammation.

In the plant, the enzyme myrosinase, a thioglucoside glucohydrolase (E.C. 3.2.1.147), is physically separated from its substrate glucosinolates, yet may be present in the same cells and tissues.^2^ When plants containing glucosinolates are subjected to mechanical disruption, myrosinase comes into contact with the glucosinolates and, depending on substrate chemistry, pH, presence of Fe^2+^ ions, and presence of countervailing enzymes such as epithiospecifier protein, converts them into either ITCs, thiocyanates, or nitriles.^3^ In animals, the conversion of dietary glucosinolates to ITCs is also carried out by the gastrointestinal microflora.^4^

**Figure 1:**
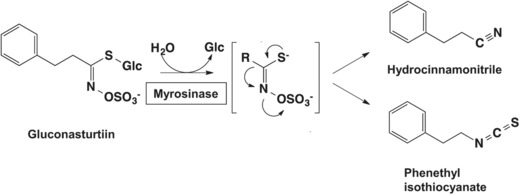
The myrosinase reaction. The glucosinolate gluconasturtiin is hydrolytically cleaved by the enzyme myrosinase to give an unstable aglycone and liberate glucose. The reaction product spontaneously rearranges into hydrocinnamonitrile and phenethyl isothiocyanate (PEITC).

**Figure 2:**
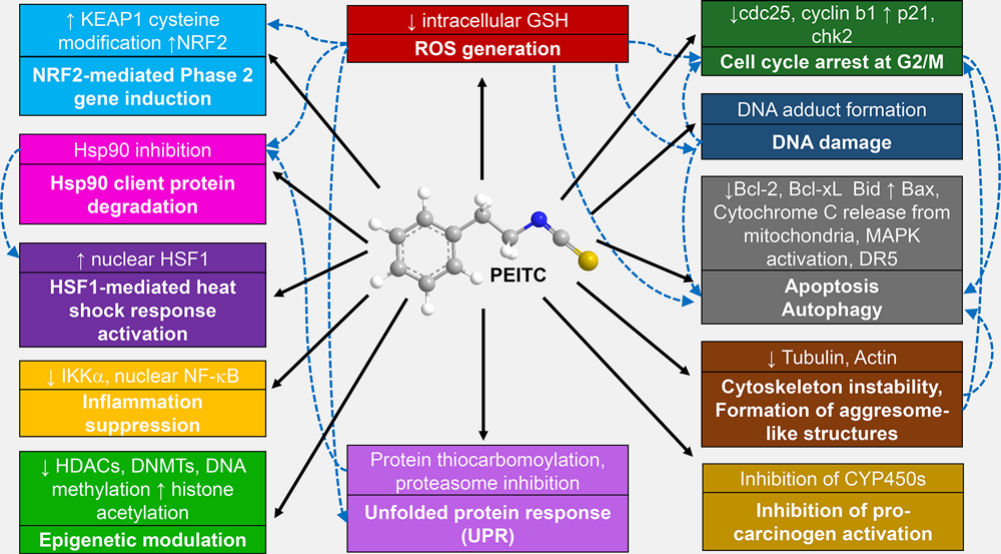
Cellular processes and pathways that are induced by PEITC (represented by the solid arrows). The dashed arrows represent the downstream effects/pathways induced as a consequence.

*Nasturtium officinale* (watercress) is a cruciferous vegetable that is rich in gluconasturtiin, the aromatic glucosinolate precursor of phenethyl isothiocyanate (PEITC; 2‐phenyl ethyl ITC; phenyl ethyl ITC; **Figure** 1).^5^ Watercress also contains smaller quantities of 7‐methylsulfinylheptyl GS and 8‐methylsulfinyloctyl GS.^6^ The seeds of the cruciferous *Barbarea verna* (land cress) are enriched with only one glucosinolate, gluconasturtiin, containing approximately 3% by weight.^7^ PEITC is one of the ITCs that have been extensively studied, and is currently in two clinical trials: for evaluating its safety and efficacy profile in head and neck cancer patients (NCT03034603), and for its long‐term effects in cancer patients’ outcomes (NCT02468882; http://www.clinicaltrials.gov). Due to the presence of the electron withdrawing ITC (N = C = S) group (Figure 1), PEITC is highly cysteine reactive and consequently has multiple intracellular targets. PEITC has been shown to induce the transcription factor nuclear factor erythroid 2 p45‐related factor 2 (NRF2)‐mediated cytoprotective pathway and the transcription factor heat shock factor 1 (HSF1)‐regulated heat shock response (HSR), inhibit phase 1 enzymes, suppress inflammation, and at high concentrations, to cause cell cycle arrest and apoptosis, alter cytoskeletal structures and the epigenome, and induce the unfolded protein response (UPR) and autophagy (**Figure** 2). The pharmacokinetics and metabolism as well as the chemopreventive and chemotherapeutic effects of PEITC have been comprehensively reviewed.^8^ Here, we focus on the ability of PEITC to induce cytoprotective pathways through activation of the transcription factors NRF2 and HSF1, and the recently described epigenetic modifications caused by this ITC. Finally, we point out that at high concentrations, PEITC causes accumulation of reactive oxygen species (ROS), leading to cytotoxicity.

## 2 PEITC Activates the NRF2‐Dependent Cytoprotective Pathway

Oxidation–reduction reactions play a central role in numerous biological processes. Living organisms are constantly exposed to ROS (e.g., superoxide anion, hydrogen peroxide, hydroxyl anion, and singlet oxygen) or reactive nitrogen species (RNS; e.g., nitroxyl anion, peroxyl nitrate, nitric oxide, and nitrosyl cation) produced by both endogenous and exogenous sources. Oxidative stress is defined as the imbalance between oxidants and antioxidants, where the former are in excess, leading to disturbances of the intracellular redox homeostasis and subsequent damage.^9^ Examples of exogenous oxidative stressors are electrophilic molecules, carcinogens such as DNA‐damaging agents, heavy metals, and ultraviolet (UV) radiation. Endogenous stressors are usually produced by intracellular metabolic reactions during processes such as respiration and inflammation. Exposure to ROS/RNS or other chemically reactive species can damage cellular macromolecules such as DNA, proteins, and cytoskeletal structures. As a consequence, chronic oxidative stress has been implicated in cancer,^10^ diabetes,^11^ neurodegenerative,^12^ respiratory,^13^ cardiovascular,^13^, ^14^ and inflammatory^15^ diseases as well as aging.^16^ In order to protect themselves from such insults, eukaryotic cells have developed several complex mechanisms to restore cellular redox homeostasis. One such mechanism is by inducing the production of antioxidant and cytoprotective proteins. This cytoprotective response is orchestrated by the transcription factor NRF2.

At basal conditions, NRF2 is negatively regulated by Kelch‐like (ECH)‐associated protein 1 (KEAP1)^17^ which functions as a substrate adaptor protein for Cullin 3–RING (really interesting new gene)‐box protein (Rbx) 1‐based E3 ubiquitin ligase, and uses a cyclical mechanism to continuously target NRF2 for ubiquitination and proteasomal degradation.^18^ Small molecules, including the ITCs sulforaphane (SFN) and PEITC, which activate NRF2 (termed inducers) block this cycle by modifying reactive cysteine sensors in KEAP1^19^ or disrupting the KEAP1–NRF2 interaction.^20^ Consequently, NRF2 is not degraded and free KEAP1 is not regenerated. The newly synthesized NRF2 accumulates, translocates to the nucleus, and binds (as a heterodimer with a small MAF protein) to antioxidant response elements (ARE, 5ʹ‐TGACnnnGC‐3ʹ) in the upstream regulatory regions of its target genes.18b–d In addition to KEAP1, the activity of NRF2 is also known to be negatively regulated by glycogen synthase kinase 3 (GSK3)/β‐transducin repeat‐containing protein 1 (β‐TrCP1)‐dependent ubiquitination and proteasomal degradation,^21^ by interaction with retinoid X receptor (RXR)α,^22^ as well as by the ubiquitin ligases synoviolin (Hrd1)^23^ and WDR23‐DDB1‐Cul4.^24^

NRF2 mediates the transcription of numerous detoxification and antioxidant genes. Examples of some of its targets are NAD(P)H‐quinone oxidoreductase 1 (NQO1), glutathione S‐transferases (GSTs), aldo‐keto reductases (AKRs), aldose reductase (AR), γ‐glutamyl peptidase (GGT), carboxylesterase (CES), heme oxygenase 1 (HO‐1), UDP‐glucuronosyltransferases (UGTs), thioredoxin (TXN), and glutamate‐cysteine ligase catalytic/modifier subunits (GCLC/GCLM).18d In human lymphoid cells, using chromatin‐immunoprecipitation (ChIP) sequencing, Chorley and colleagues identified 242 high confidence NRF2 binding sites upon treatment with SFN.^25^ The coordinate upregulation of these NRF2 targets provides the cell with long‐lasting protection against a plethora of damaging insults,^26^ allowing adaptation and survival and restoring homeostasis.

Numerous reports have documented the cytoprotective role of NRF2 activation by PEITC, and these have been recently reviewed.8a–e Several recent studies after the publication of these reviews are noteworthy. Through activation of NRF2, PEITC restored insulin‐stimulated glucose uptake, translocation of glucose transporter 4, and insulin signaling that were impaired by treatment of 3T3‐L1 adipocytes with hydrogen peroxide,^27^ demonstrating the cytoprotective role of PEITC under conditions of dysregulated glucose metabolism. Dietary administration of PEITC reduced mucosal and submucosal inflammation, glandular atypia, and tumor development in a mouse model of colitis‐associated colorectal carcinogenesis.^28^ PEITC was also suggested as one potential dietary agent for the prevention and treatment of malignant mesothelioma.^29^ Treatment with PEITC of neonatal humanized UDP‐glucuronosyltransferase 1 (hUGT1) mice, which have high levels of serum bilirubin due to a developmental delay in the expression of the NRF2 transcriptional target UGT1A1, the enzyme that catalyzes the conjugation of bilirubin, induced UGT1A1 in liver and intestine, and reduced the serum bilirubin.^30^ In addition to upregulating the transcription of cytoprotective genes, NRF2 downregulates the expression of genes involved in lipid synthesis, including acetyl‐CoA carboxylase 1 (ACC1), fatty acid synthase (FASN), and carnitine palmitoyltransferase 1 (CPT1),^31^ and this downregulation has been proposed to play a role in the ability of PEITC to prevent prostate cancer in the transgenic adenocarcinoma of mouse prostate (TRAMP) model.^32^ In human prostate cancer cells, exposure to PEITC induced the appearance of membranous vacuoles and the recruitment of microtubule‐associated protein 1 light chain 3 (LC3) to autophagosomes, promoting autophagy.^33^ Interestingly, inhibition of autophagy enhanced the inhibitory effect of PEITC on the metastatic potential of lung cancer cells,^34^ suggesting that the PEITC‐mediated autophagy plays a cytoprotective role. NRF2 activation promotes autophagic flux,^35^ but whether or not NRF2 is required for the ability of PEITC to induce autophagy, has not been examined.

**Figure 3:**
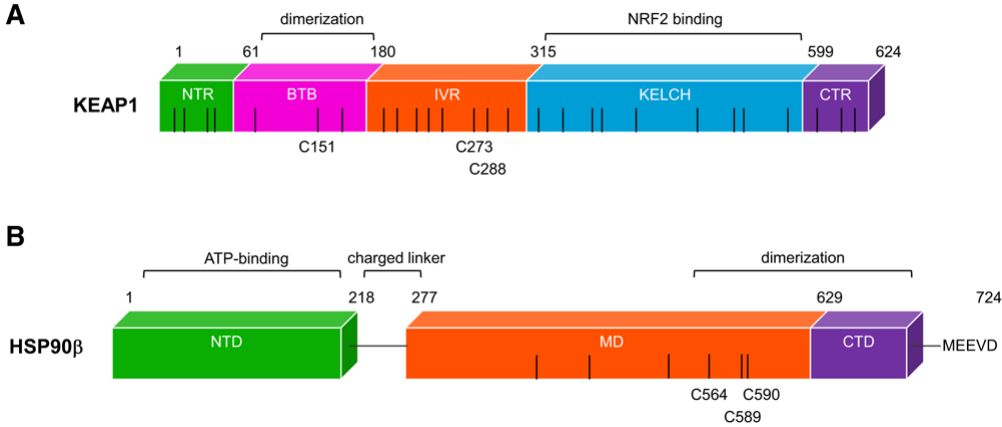
Schematic representation of mouse KEAP1 and human HSP90β. A) KEAP1 is a homodimeric protein which has five distinct domains: N‐terminal region (NTR), broad complex, Tramtrack, Bric‐á‐brac (BTB), intervening region (IVR), Kelch domain (KELCH), and the C‐terminal region (CTR). B) HSP90β has an N‐terminal domain (NTD), where ATP binds. The middle domain (MD) allows for client protein binding and the C‐terminal part of the MD together with the C‐terminal domain (CTD) allows for homodimerization of the chaperone. Various co‐chaperones are able to bind to all three domains with different affinities. Client proteins are also able to interact with each of the HSP90 domains. The black bars represent cysteine residues present in each of the proteins, and some of the reactive cysteines are indicated.

**Figure 4:**
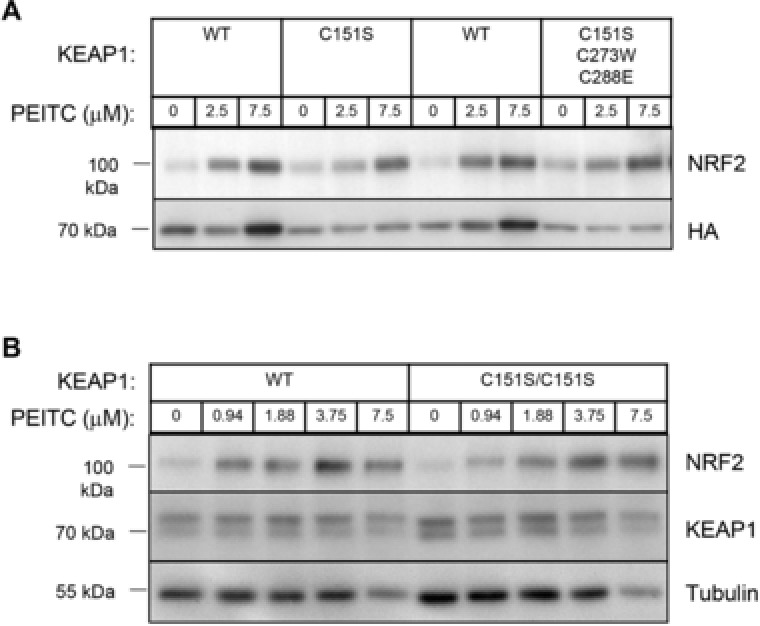
C151 in KEAP1 is the primary sensor for PEITC, but is not required at high inducer concentrations for the stabilization of NRF2. A) Stable KEAP1‐knockout MEF cells rescued with either WT, single mutant C151S, or triple mutant C151S/C273W/C288E of mouse N‐terminally tagged HA‐KEAP1 were generated as described.^38^ Cells were plated in 6‐well plates at a density of 10^6^ cells per well, and placed in a 37 °C humidified incubator in 5% CO_2_ in air. On the following day, cells were treated with vehicle (0.1% DMSO) or the indicated concentrations of PEITC for 3 h. B) The animal experiments were approved by the Tohoku University Animal Care Committee and were compliant with the regulations of The Standards for Human Care and Use of Laboratory Animals of Tohoku University (Sendai, Japan) and the Guidelines for Proper Conduct of Animal Experiments of the Ministry of Education, Culture, Sports, Science and Technology of Japan. Wild‐type and KEAP1^C151S/C151S^ knock‐in mice were generated, bred, and maintained at Tohoku University. Peritoneal macrophage production was elicited by i.p. injections of 4% thioglycolate solution using the previously described method.^38^ Four days later, peritoneal macrophage cells were extracted, washed, plated in 6‐well plates at a density of 10^6^ cells per well, and placed in a 37 °C humidified incubator in 5% CO_2_ in air. Four hours later, when the cells had adhered to the plates, they were washed twice with phosphate buffered saline (PBS) before proceeding with treatments with vehicle (0.1% DMSO) or the indicated concentrations of PEITC for 3 h. For western blot analysis, cells were lysed, proteins were separated by electrophoresis on an 8% SDS‐polyacrylamide gel, and electrophoretically transferred to a polyvinylidene difluoride (PVDF) membrane. After blocking with 10% nonfat milk at room temperature for 1 h or overnight at 4 °C, immunoblotting was performed using the following antibodies for either 1–2 h at room temperature or overnight at 4 °C: rat monoclonal NRF2 antibody [154] at a dilution of 1:100, rat monoclonal HA antibody (Roche, 3F10, CA, USA, at a dilution of 1:1000) or rat monoclonal KEAP1 antibody [154] at a dilution of 1:100. A mouse monoclonal antibody against α‐tubulin (Sigma‐Aldrich, DM1A, 1:5000–1:10000 dilution) was used as a loading control.

Due to its highly reactive electron withdrawing isothiocyanate group (Figure 1), PEITC reacts readily with the cysteine of glutathione in a reaction catalyzed by glutathione transferases,^36^ and it has been assumed that the ITC activates NRF2 by modifying cysteine residue(s) in KEAP1. However, the precise identity of the cysteine sensor(s) in KEAP1 for PEITC is unknown. This is an important question in view of the prominent role of NRF2 as a mediator of the cytoprotective activity of PEITC. Amongst the 25 (mouse) or 27 (human) cysteines in KEAP1, C151, C273, and C288 are the best‐characterized inducer sensors (**Figure** 3). It is established that C151 serves as the main sensor cysteine in KEAP1 for SFN.^37^ To identify the cysteine sensor(s) for PEITC, we used stable cell lines of KEAP1‐knockout mouse embryonic fibroblast cells (KKO MEF cells) that had been rescued with either wild‐type (WT) KEAP1 or the KEAP1 mutants C151S and C151S/C273W/C288E by introduction of an hemagglutinin (HA)‐tagged KEAP1 expression vector ligated to the PiggyBac transposon system.^38^ Importantly, all of these mutants have been previously designed and validated to retain their ability to target NRF2 for degradation.^38^ Exposure to PEITC caused stabilization of NRF2 in cells from all genotypes. A closer inspection revealed that compared to KKO MEF cells rescued with the WT KEAP1, NRF2 stabilization following exposure to the low (2.5 μm) concentration of PEITC was attenuated in the KKO MEF cells rescued with either the single mutant C151S or the triple mutant C151S/C273W/C288E KEAP1 (**Figure** 4A), indicating that C151 plays a role in the sensing mechanism. However, exposure to the high (7.5 μm) concentration of PEITC caused the stabilization of NRF2 to nearly identical levels in all genotypes regardless of the mutation status of KEAP1 (Figure 4A). Curiously, for reasons that are unclear at this time, at the high concentration tested, PEITC increased the levels of HA‐KEAP1 only in cells expressing wild type, but not any of the mutants of KEAP1.

We had previously generated KEAP1^C151S/C151S^ knock‐in mice using the CRISPR/Cas9 gene editing technology.^38^ This model provided an opportunity to test the importance of C151 in sensing of PEITC using primary cells from these mice. Primary peritoneal macrophages can be obtained in high yield and purity after sterile elicitation with thioglycolate broth^39^ and are very sensitive to NRF2 activation ex vivo.^40^ We therefore used this experimental system to compare NRF2 stabilization by PEITC (at concentrations ranging from 1 to 7.5 μm) in primary peritoneal macrophage cells isolated from wild‐type and KEAP1^C151S/C151S^ knock‐in mice. In close agreement with the data in MEF cells, we found that following treatment with the lowest (1 μm) concentration of PEITC, the stabilization of NRF2 was attenuated in the KEAP1^C151S/C151S^ primary macrophage cells compared to their wild‐type counterparts (Figure 4B). However, this difference between the genotypes was lost when the cells were exposed to higher concentrations of PEITC. Notably, lower concentrations of PEITC were used for the experiments with primary macrophage cells than the concentrations used for MEF cells due to the greater inducer sensitivity of primary cells compared to established cell lines.

Together, these data strongly suggest that C151 in KEAP1 is the primary sensor for PEITC, but at high concentrations of the ITC, this cysteine becomes “saturated,” and other cysteines are also modified, ultimately leading to NRF2 stabilization. Notably, C151 in KEAP1 is particularly highly reactive due to the presence of five proximate basic amino acid residues (H129, K131, R135, K150, and H154),^41^ which have the ability to deprotonate the sulfhydryl group of the cysteine, thus lowering its p*K_a_*. These data also raise the possibility that, at high inducer concentrations, cysteine(s) in other proteins may react with PEITC. It is interesting to note that the highest concentration of PEITC (7.5 μm) caused a significant reduction in the protein levels of α‐tubulin (Figure 4B). It has been reported that the ITCs benzyl‐ITC (BITC), PEITC, and SFN covalently modify α‐tubulin in cells by reacting with its cysteine residues and as a consequence, preventing its polymerization^42^ (also see section 5 below). In addition, PEITC promotes the degradation of both α‐ and β‐tubulin in prostate cancer PC3 cells; this degradation could be rescued by co‐incubation with the antioxidant N‐acetyl‐L‐cysteine (NAC).^43^ Together, these results confirm that cysteine reactivity underlies the multiple biological activities of PEITC.

## 3 PEITC Activates the HSF1‐Dependent Heat Shock Response

The HSR is a transcriptional program that is elicited by the transcription factor HSF1 which in normal cells is a cytoprotective mechanism to ensure adaptation and survival during conditions of stress, including exposure to elevated temperatures or proteotoxic chemicals, oxidative stress, heat shock protein 90 (HSP90) inhibition, and proteasome inhibition. We have shown previously that numerous sulfhydryl‐reactive NRF2 activators, are able to activate the HSR through the transcription factor HSF1, although the concentrations required for HSF1 activation are higher than those that activate NRF2.^44^ Over the past decade, a number of studies have shed light onto the capacity of ITCs to induce the HSR. For example, SFN causes nuclear accumulation of HSF1^45^ and induces the expression of HSP27 and HSP70.^45^, ^46^ BITC and PEITC have also been reported to induce the expression of heat shock proteins^47^ and reduce the formation of aggresome‐like structures following proteasomal inhibition.^48^

In one proposed mechanism, under basal conditions, the inactive monomeric HSF1 is primarily sequestered in the cytoplasm by the HSP90 multi‐chaperone complex.^49^ The HSP90 multi‐chaperone complex includes co‐chaperone proteins p23, HSP70, HSP40, scaffold for HSP90/HSP70 interaction (STIP1), and cell division cycle 37 (CDC37).^50^ HSP90 mediates the folding and stabilization of its client proteins through its ATPase cycle, which is mediated by dynamic interactions with its co‐chaperones. Inhibition of HSP90 function by pharmacological inhibitors can manifest in the following ways: 1) physical binding to HSP90 itself at its N‐terminal ATP binding pocket or at its C‐terminus, 2) modification of the cysteines which are only present in its middle domain, 3) physical interaction with its co‐chaperones, and 4) global increase in the levels of unfolded proteins. In all the four circumstances, HSP90 is unable to mediate its client protein folding and stabilization. HSP90 has more than 200 client proteins.^51^ Exposure to stress causes HSF1 to dissociate from the HSP90 multi‐chaperone complex, and promotes its hyperphosphorylation. Subsequently, HSF1 translocates to the nucleus where it forms transcriptionally active homotrimers that bind to genomic heat shock element (HSE) sequences (5ʹ‐nTTCnnGAAn‐3ʹ) to activate transcription of its diverse target genes that encode proteins involved in molecular folding and chaperoning, metabolism, detoxification, and cell signaling.^52^ HSF1's transcriptional activation is attenuated by binding to HSP90, HSP70, and HSP40, all of which are its transcriptional targets, forming a negative feedback regulatory loop.^53^

Due to the mounting evidence that the ITCs induce heat shock proteins, we tested a series of mildly electrophilic SFN analogues, and found that sulfoxythiocarbamate alkyne (STCA) modifies cysteines in HSP90 (Figure 3).^54^ We also found that such sulfoxythiocarbamate derivatives, by virtue of their sulfhydryl reactivity, destabilize HSP90 client proteins and induce the HSR.^55^ With this knowledge, we studied the ability of PEITC to inhibit HSP90.^56^ We found that PEITC causes the dissociation of HSF1 from HSP90 and the nuclear accumulation of the transcription factor.^56^ In cervical cancer HeLa cells stably transfected with the HSP70.1 promoter fused upstream of the luciferase gene, PEITC robustly induced the promoter activity in a time‐ and dose‐dependent manner.^56^ HSP70, the prototypic marker of activation of the HSF1‐mediated transcription, was induced by PEITC and the induction was absent in MEF cells deficient in HSF1, suggesting that PEITC induced the HSR by activating HSF1.^56^ In agreement with our findings, PEITC treatment in murine hepatoma Hepa1c1c7 cells induced the HSP70 gene expression.47a Furthermore, PEITC caused the degradation of classical HSP90 client proteins human epidermal growth factor receptor 2 (HER2) and Raf‐1 proto‐oncogene, serine/threonine kinase (RAF1) in MDA‐MB‐231 breast cancer cells.^56^ This is consistent with a number of publications reporting that exposure to PEITC decreases the levels of proteins such as CDC25, CYCLIN B1, cyclin‐dependent kinase 1 (CDK1), as well as several histone deacetylases (HDACs), DNA methylases (DNMTs), and matrix metalloproteinases (MMPs), all of which are HSP90 client proteins (**Table** 1). Another HSP90 client protein is the pleiotropic cytokine macrophage migration inhibitory factor (MIF), which is often elevated in cancer cells and correlates with tumor aggressiveness and poor prognosis.^57^ Curiously, PEITC covalently modifies MIF at its N‐terminal proline, P2, causing conformational changes which disrupt its catalytic tautomerase activity as well as its binding to other proteins.^58^

**Table 1:** Examples of HSP90 client proteins that are downregulated at the protein level in response to PEITC categorized by their associated roles

Function	HSP90 Clients
Cell division cycle	CDC25,^90^ CYCLIN D1,^91^ CYCLIN B1,^92^ CDK1,^90^, ^92^ CDK4,^92^ CDK6,^92^ WEE174d
Apoptosis	BCL‐2,^80^, ^92^ BCL‐xL^80^, ^81^
Angiogenesis	HIF‐1α,^93^ VEGFR1^94^
Invasion and metastasis	MMP2, 9^95^
DNA damage response	SP1,3,4,^94^ DNMT1, 3A, 3B^66^
Proliferation and survival	HER2,^56^, ^96^ STAT3,^96^ RAF1,^56^ EGFR,^96^ AKT,^70^, ^97^ PDK195a

Phosphorylation of HSF1 at serine 326 (pS326) is one of the primary and hallmark posttranslational modification (PTM) events that occur during HSF1 activation. Within minutes, PEITC induced the phosphorylation of HSF1 at S326 as well as the activation of the p38 mitogen‐activating protein kinases (MAPKs).^56^ By the use of p38 MAPK inhibitors, we found that the p38 gamma isoform was, in part, responsible for the phosphorylation of HSF1 pS326 upon PEITC exposure. A higher concentration of PEITC caused a persistent phosphorylation at S326 and activation of the HSR even 24 h after treatment. In many cancers, HSF1 is activated and it has been shown that HSF1 overexpression promotes aneuploidy and malignant phenotypes as cells become addicted to the cytoprotection it confers.^59^ Specifically, high HSF1 pS326 levels correlate with poor patient prognosis in several cancers.^60^ Cancer cells have elevated HSF1 and heat shock protein levels due to the increased levels of mutant proteins, which require stabilization through these chaperoning machineries. Therefore, in the context of cancer, inhibiting the “normal” HSP90 function is beneficial; however, the consequent activation of the HSF1‐mediated transcriptional response feeds these oncogenic protein helpers (i.e., HSP90 and HSP70) into the same system. Therefore, one of the challenges in targeting HSP90 in cancer is to find compounds that specifically inhibit HSP90 without activating the HSR, or to use classical HSP90 inhibitors in combination with HSF1 inhibitors.

It is currently not known how PEITC inhibits HSP90, and several possibilities could be considered. First, similar to STCA, PEITC may directly modify cysteines in HSP90. Second, PEITC may modify cysteines in HSP70. By use of [^14^C]‐PEITC and mass spectrometry, PEITC has been found to bind HSP70, which is a component of the HSP90 multi‐chaperone complex.^61^ Third, PEITC has been reported to induce the UPR^62^ and inhibit the 20S and 26S proteasomal subunits,^63^ where both of these conditions have been shown to induce the HSF1‐mediated HSR.

## 4 PEITC and Epigenetic Regulation: An Emerging Field

In the prostate cancer cell line LNCaP, the *GSTP1* gene is silenced due to the aberrant methylation of its promoter. In these cells, PEITC exposure induced demethylation of the CpG island of the *GSTP1* promoter, and ultimately reactivated *GSTP1* expression.^64^ In this study, PEITC was shown to decrease the activity of HDAC1 and HDAC2, and decrease the expression of the HDAC1. Also, PEITC promoted the acetylation of histone H3 (hH3), methylation of hH3 at lysine 4, and the demethylation of hH3 at lysine 9. Remarkably, the androgen responsive LNCaP cells when treated with PEITC were able to differentiate into prostate cancer stem cells (PCSC) floating spheres, leading the researchers to hypothesize the epigenetic alterations exerted by ITC, decreased expression of DNMT1, increased histone H3K4 acetylation, and reactivated GSTP1 expression in the spheric PCSCs.^65^ Interestingly, PEITC treatment in LNCaP cells causes the epigenetic reactivation of Ras‐association domain family 1 isoform A (*RASSF1A*) gene by enhancing CpG demethylation at its promoter. In this study, PEITC also reduced the expression of the DNMTs 1, 3A, and 4 as well as HDACs 1, 2, 4, and 6.^66^ Additionally, in LPS‐stimulated human colon epithelial cells SW480, PEITC treatment decreased the expression of several genes (e.g., interleukin 8 [IL‐8], MMP7, signal transducer and activator of transcription 1 [STAT1], and nuclear factor of kappa light chain gene enhancer in B cells 1, p105 [NF‐κB1]) by causing the trimethylation of hH3 at lysine 27 (H3K27me3).^67^ It is yet to be investigated whether the histone methylase(s) that is involved in the trimethylation at H3K27me3 is in some way activated by PEITC treatment.

Recently, Lawson and colleagues found that, possibly through the modification of their cysteines, PEITC targets various deubiquitinases (DUBs) many of which (e.g., VCPIP1, USP1, USP3, USP16, and USP48) are involved in chromatin remodeling and DNA repair. PEITC also decreases the levels of DUBs by initiating their degradation.^68^ Another mechanism by which PEITC could regulate the epigenome is by altering the expression of a number of microRNAs (miRNAs).^69^ In PC3 cells, PEITC induced the expression of miR‐194, which was able to suppress cell invasiveness in vitro. In the same system, miR‐194 induction by PEITC inhibited the expression of bone morphogenetic protein 1 (BMP1) which in turn attenuated the expression of the oncogenic MMP2 and MMP9. Suppression of MMP9 expression (by inhibiting NFκB and AP‐1) and reduction of lung metastasis by PEITC, BITC, and SFN was shown in an A549 xenograft mouse model.^70^ The inhibition of MMP2 activity by PEITC and BITC, which was accompanied by reduced cell migration and invasion, was also reported in the human melanoma cell line A375.S2^71^ and in the mouse melanoma cell line B16F10.^72^ Both MMP2 and MMP9 are client proteins of HSP90 (Table 1), but whether or not the inhibitory effect of PEITC could be in part a consequence of HSP90 inhibition has not been investigated. Most recently, it was found that in human prostate cancer cells (LNCaP and PC3), PEITC induced the expression of the epigenetic regulator SET domain containing lysine methyltransferase 7 (Setd7), which in turn activated NRF2, providing an epigenetic mechanistic link to enhancing NRF2‐dependent cytoprotection by the ITC.^73^

## 5 At High Concentrations, PEITC is Cytotoxic

Inhibition of HSP90 and/or glutathione depletion, leading to the accumulation of ROS, all of which are related to the cysteine reactivity of PEITC, may provide potential mechanisms for the observation that, like other ITCs, high concentrations of PEITC are cytotoxic. PEITC arrests cells at the G2/M phase.^74^ In DU145 prostate cancer cells, this cell cycle arrest was mediated by an increase in p53, and a decrease in CDC25C protein levels.^74^ ^d]^ PEITC activation of p53 in oral squamous cell carcinoma cell lines with a functional p53 caused an induction of the ataxia telangiectasia mutated serine/threonine kinase‐checkpoint kinase 2 (ATM‐CHK2) pathway as well as the expression of p21. Interestingly, in breast cancer cells expressing mutant p53, PEITC exposure caused the depletion of mutant p53.^75^ Exposure to PEITC has also been reported to reduce the levels of other cell cycle regulating proteins such as CYCLIN B1 and CDK1 contributing to the cell cycle arrest at G2/M.^76^ Since mutant p53, CYCLIN B1, and CDK1 are HSP90 client proteins (Table 1), it is possible that inhibition of HSP90 is an important contributor for their downregulation upon treatment with PEITC.

Additionally, PEITC induces extrinsic and intrinsic apoptotic pathways in cancer cells^77^ by promoting ROS production,^77^, ^78^ activating the MAPKs pathways,^79^ downregulating the anti‐apoptotic proteins Bcl‐2 and Bcl‐x,^80^ and activating the pro‐apoptotic proteins Bax and Bak.^81^ Exposure to PEITC causes the activation of stress‐activated MAPKs such as c‐Jun N‐terminal kinase (JNK),77a p38 MAPKs,^56^, ^82^ and extracellular signal‐regulated kinase1/2 (ERK1/2).^83^ It has been shown by several groups that the activation of these kinases by PEITC correlates with activation of the caspase‐dependent apoptotic pathway, and that chemical inhibition of these kinases blocks this process. For example, chemical inhibition of only JNK activity, and not p38 MAPK or ERK1/2, in human colon adenocarcinoma cells (HT‐29) reduced PEITC‐induced apoptosis.79a Inhibition of the p38 MAPK with the inhibitor SB202190 did not prevent apoptosis activation by PEITC in PC3 cells; however, treatment with the ERK1/2 inhibitor, PD98059 achieved this effect.^82^ In another study in human oral cancer cells (HN22), inhibition of p38 MAPK with the SB203580 p38 MAPK inhibitor blocked apoptosis mediated by PEITC's activation of the death receptor 5 (DR5).^84^ Furthermore, it has been shown that compared to normal cells, cancer cells are more sensitive to PEITC‐mediated cell death.^85^ The seemingly conflicting evidence on the dependence of apoptosis on PEITC‐induced activation of specific MAPKs could be attributed to differences in the cancer cell lines used in these studies, including their individual MAPK and caspase status, and apoptotic susceptibility.

Another mechanism by which PEITC may promote cell death is through its ability to modify cellular cytoskeletal elements. Human lung cancer cells (A549) treated with radiolabelled PEITC or SFN exhibited binding of the ITCs to tubulin.^86^ ITCs cause tubulin depolymerization in vitro and in vivo by covalently binding to its cysteines.^86^ Because A549 cells have constitutively active NRF2 due to inactivating mutations in KEAP1,^87^ this finding highlights the importance of sulfhydryl reactivity of the ITCs in covalently modifying cysteine residues even under conditions of constitutive NRF2 activation. PEITC also inhibited the expression of α‐ and β‐tubulin in PC3 cells.^43^ The modification and depolymerization of tubulin by PEITC might be part of the mechanism by which the ITC inhibits cell proliferation and induces apoptosis and cell cycle arrest. In addition, the covalent modification of α‐ and β‐tubulin by PEITC has been shown to induce the formation of aggresome‐like structures.47e In the breast cancer cell lines MCF7 and MDA‐MB‐231, a 48 h exposure of PEITC‐induced α‐tubulin acetylation, possibly through the inhibition of HDAC6.^88^ In the lung cancer cell lines A549 and H1299, in addition to tubulin degradation, PEITC caused disassembly of actin stress fibers, accompanied by cytotoxicity, which was less pronounced in A549 cells,^89^ in agreement with their constitutively high NRF2 levels.

## 6 Conclusion

PEITC has multiple intracellular targets. PEITC activates the cytoprotective transcription factors NRF2 and HSF1 and modifies the epigenome. At high concentrations, PEITC is cytotoxic. Importantly, the ability to react with cysteines has been implicated in all of the biological activities of PEITC. Hence, the on‐target selectivity of PEITC depends on its concentration. It is thus essential to carefully consider the dose and duration of treatment when designing future clinical trials.

## Conflict of interest

The authors declare no conflict of interest.
